# The Challenge of E-Spinning Sub-Millimeter Tubular Scaffolds—A Design-of-Experiments Study for Fiber Yield Improvement

**DOI:** 10.3390/polym16111475

**Published:** 2024-05-23

**Authors:** Cilia A. Sandhoff, Alexander Loewen, Yasmin Kuhn, Haude-Tukua Vidal, Stephan Ruetten, Stefan Jockenhoevel

**Affiliations:** 1 Department of Biohybrid & Medical Textiles (BioTex), AME Institute of Applied Medical Engineering, Helmholtz Institute, RWTH Aachen University, Forckenbeckstr. 55, 52074 Aachen, Germany; sandhoff@ame.rwth-aachen.de (C.A.S.); loewen@ame.rwth-aachen.de (A.L.); kuhn@ame.rwth-aachen.de (Y.K.); 2 Laboratoire de Physique et Mécanique Textiles (LPMT), École Nationale Supérieure d’Ingénieurs Sud-Alsace, 12 Rue des Frères Lumière, 68 093 Mulhouse, France; 3 Electron Microscopy Facility, University Hospital Aachen, Pauwelsstr. 30, 52074 Aachen, Germany; sruetten@ukaachen.de; 4 Aachen-Maastricht Institute for Biobased Materials (AMIBM), Maastricht University, Urmonderbaan 22, 6167 RD Geleen, The Netherlands

**Keywords:** electrospinning, PLGA, tissue engineering, sub-millimeter tubular scaffold

## Abstract

In tissue engineering, electrospinning has gained significant interest due to its highly porous structure with an excellent surface area to volume ratio and fiber diameters that can mimic the structure of the extracellular matrix. Bioactive substances such as growth factors and drugs are easily integrated. In many applications, there is an important need for small tubular structures (I.D. < 1 mm). However, fabricating sub-millimeter structures is challenging as it reduces the collector area and increases the disturbing factors, leading to significant fiber loss. This study aims to establish a reliable and reproducible electrospinning process for sub-millimeter tubular structures with minimized material loss. Influencing factors were analyzed, and disturbance factors were removed before optimizing control variables through the design-of-experiments method. Structural and morphological characterization was performed, including the yield, thickness, and fiber arrangement of the scaffold. We evaluated the electrospinning process to enhance the manufacturing efficiency and reduce material loss. The results indicated that adjusting the voltage settings and polarity significantly increased the fiber yield from 8% to 94%. Variations in the process parameters also affected the scaffold thickness and homogeneity. The results demonstrate the complex relationship between the process parameters and provide valuable insights for optimizing electrospinning, particularly for the cost-effective and reproducible production of small tubular diameters.

## 1. Introduction

Small tubular scaffolds in the sub-millimeter range are highly needed in the development of medicine products, e.g., in nerve [1,2], islet cell [3], and cardiovascular tissue engineering [4]. Within tissue engineering, electrospinning has gained significant attention owing to its ability to create highly porous structures characterized with an exceptional surface area to volume ratio and fiber diameters that can mimic the structure of the extracellular matrix (ECM). The porous nature of these non-woven electrospun structures significantly aids in facilitating nutrient exchange. Using biodegradable materials, the scaffold can act as a drug delivery system for bioactive substances, thereby promoting cell growth, differentiation, and specific tissue formation.

Electrospinning (e-spinning) is already being successfully established to produce functional bioactive scaffolds and guiding structures in tissue engineering [5,6,7,8,9]. Although there is a clear need of sub-millimeter tubular structures, most literature deals with the production of larger diameters, e.g., in vascular tissue engineering 1.5–80 mm [10,11,12,13]; neural tissue engineering 1.26–100 mm [14,15]; intestinal tissue engineering 2.5–32 mm [16,17]; and tracheal tissue engineering 4–20 mm [18,19]. Therefore, there is still a lack of process understanding for sub-millimeter collector structures.

Many studies have been conducted to investigate the effects of various influencing factors on fiber morphology for e-spinning in general. For example, Putti et al. [20] investigated the effect of relative humidity and temperature, which influence solution evaporation rates, subsequently impacting fiber structure. Similarly, Ruiter et al. [21] investigated the effect of polymer concentration and polymer flow rate on fiber properties. Furthermore, it was found that control factors including electrode arrangement, applied voltage, polymer solution conductivity and viscosity, and the collector influence the electric field [20]. Electrode positioning and arrangement directly shape the fibers, while different types of electrodes and their distances affect electric field homogeneity [22]. The applied voltage between the nozzle and the collector determines the field intensity. Higher voltages lead to stronger electrostatic forces, resulting in more stretched (thinner) nanofibers [23]. Enhanced electrical conductivity in the polymer solution facilitates efficient charge transfer and the generation of electrostatic forces, improving the spinnability of the fibers. The polymer solution concentration and viscosity determine drop formation and stability, thereby affecting fiber diameters [23]. However, all of these studies were conducted using large collectors. This study aims to address the lack of knowledge regarding the effect of chosen control factors on e-spinning on sub-millimeter diameter collectors. 

**Figure 1:**
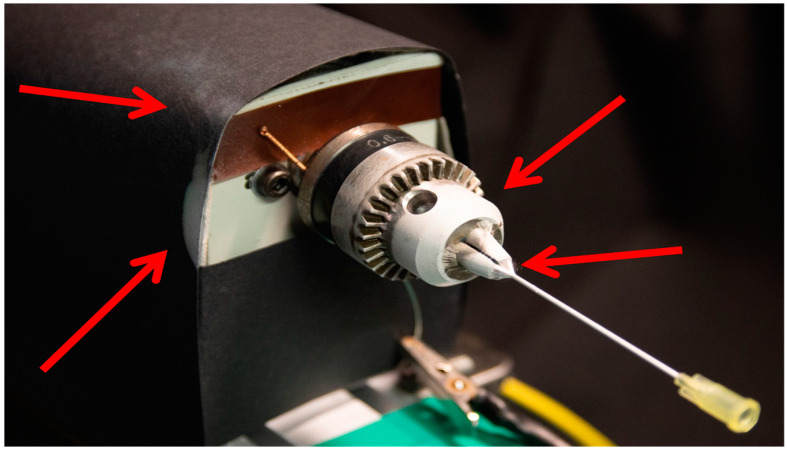
Contamination of the environment and devices with an unstable electrospinning process, leading to more than 80% material loss; red arrows mark significant material loss on the rotation device (white polymer stains).

A major challenge in sub-millimeter e-spinning is that the influence of disturbance factors on the spinning process increases significantly with the reduction in the collector size (reciprocal scale effect). If the collector becomes smaller, the directional electric spinning field between the spinning nozzle and the collector decreases and becomes more unstable. This results in reduced reproducibility and significant material loss due to process instability. In addition to further disturbance variables (see Table 1), the design of the spinning device plays a decisive role. Due to the small collector area, other conductive materials or electrical components (e.g., motors) in the immediate surrounding of the collector have an important influence on the electric field. In conventional e-spinning setups, fiber yields on the collector in the sub-millimeter level range from 5% to 15% due only to the massive loss on non-collector surfaces (Figure 1). As the incorporation of drugs and bioactive substances is more and more important and can be very expensive (1000 to 20,000 EUR per milligram), this low fiber yield is very problematic from a production point of view.

**Figure 2:**
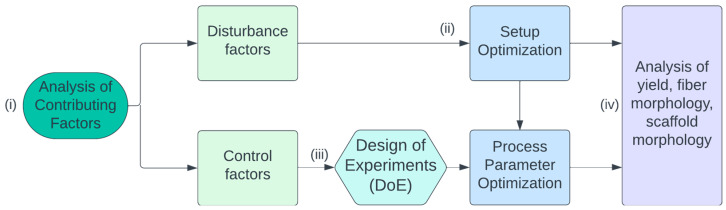
Flow chart of the study. (i) Contributing factors of e-spinning were analyzed and distinguished into disturbance and control factors. With an optimized setup (ii), a design-of-experiments study (iii) was designed and the process parameters were optimized. (iv) E-spun scaffolds were analyzed.

Therefore, we conducted a **design-of-experiments (DoE)** study in which we first **(i) analyzed the relevant influencing factors** theoretically and divided them into control and disturbance factors. This resulted in **(ii) the optimization of the experimental setup** at first in order to eliminate the disturbance factors as best as possible. Finally, **(iii) the influence of relevant control factors** was investigated experimentally on the basis of the DoE method, **and (iv) analyzed with regard to the optimum fiber yield** (Figure 2).

## 2. Materials and Methods

### 2.1. Definition Target Variables

The primary objectives of this investigation involve enhancing the fiber yield on the collector and stabilizing the e-spinning process. This includes identifying and eliminating disturbances via a novel setup design, alongside assessing the influence of control variables on the final produced tubular scaffold. To determine the fiber yield on the collector, the mass deposition of fibers onto the collector post-electrospinning was quantified. As a reliable parameter, the examination of scaffold structure (diameter and uniformity) and the assessment of fiber diameter along with fiber diameter variability are considered. Additionally, scaffold weight is analyzed to determine the obtained fiber yield on the collector (Table 1).

### 2.2. Key Parameter Analysis

Initially, the parameters affecting the e-spinning process were analyzed. Table 2 provides an overview of the factors influencing the e-spinning process, along with a brief description and classification into control and disturbance factors. Pre-studies showed the gray highlighted factors in Table 2 have a high impact on yield. These factors were examined in detail within the scope of this study. 

### 2.3. Disturbance Factors Reduction

Minimizing disturbances is a crucial preliminary step in implementing an effective e-spinning process, ensuring enhanced reproducibility and fiber quality. Before proceeding with the DoE, it is imperative to stabilize the environment and eliminate or minimize external interferences, such as the influence of the rotational motor and the laboratory-lifting platform, to prevent fiber deviation from the collector. Therefore, a new setup was built. Furthermore, the new setup enables the application of a negative voltage to the collector. The new setup is housed within a climate chamber (KBF 720, Binder, Tuttlingen, Germany) to maintain consistent humidity and temperature levels throughout the e-spinning process.

### 2.4. Optimization of Control Factors

A rating matrix was set up to select the control factors. This matrix evaluates the suitability of e-spinning control factors for the intended experiments, considering the expected effects on the target variables, the complexity of modifications, and the interdependence with other control factors. Subsequently, the total score for each control factor is determined, which serves as the basis for the selection process. From this rating matrix, three control factors were chosen for this DoE (Table 3). 

**Figure 3:**
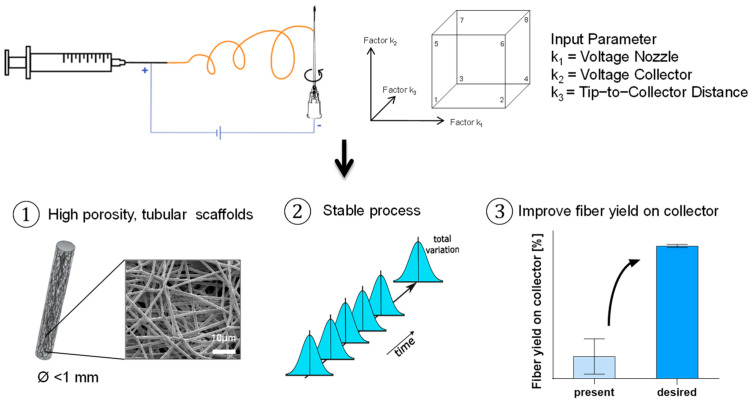
Schematic description of the design−of−experiments (DoE) study design.

A full factorial 23 DoE study was conducted to investigate the correlation of the applied voltage on the spinning nozzle and collector and the tip-to-collector distance on the solution e-spinning process of sub-millimeter tubular structures (Figure 3). The primary goal was to optimize these parameters for maximum yield. In addition to the yield, the morphology of the fibers was examined using SEM and the morphology of the scaffolds using digital microscopy.

### 2.5. Fabrication of E-Spun Tubular Scaffold

The study was carried out using Poly(lactid-co-glycolide) (PLGA) as a demonstrator polymer, due to its degradability and resulting use as a drug release material. A solution of 10 (m/V)% Poly(lactide-co-glycolide) (PLGA, Purasorb^®^ PLG 8523, 85/15 L-Lactide/Glycolide, Corbion, Amsterdam, The Netherlands) was prepared by dissolving the polymer in 1,1,1,3,3,3-Hexafluoro-2-propanol (HFIP, Sigma, Taufkirchen, Germany, purity ≥ 99%, CAS-No: 920-66-1) overnight at room temperature. Proteins and active ingredients are temporarily stable in this solvent. To spin drug-loaded scaffolds, a 10 (m/V)% PLGA solution containing 20 mg/mL Dexamethasone (Sigma, Taufkirchen, Germany, purity ≥ 97%) and a 10 (m/V)% PLGA solution containing 40 mg/mL Dexamethasone were prepared. The solutions were electrospun for 10 min from a 1 mL syringe (BD PlastipakTM, Eysins, Switzerland) with a needle gauge of 21 (Sterican^®^, B. Braun, Melsungen, Germany) and a flow rate of 0.5 mL/h. A high voltage was applied to the tip of the needle attached to the syringe (Power Charger 60 V1.5.0, Eltex-Elektrostatik-GmbH, Weil am Rhein, Germany). Depending on the run, a negative high voltage was applied to the collector (Power Charger 30 V1.5.0, Eltex-Elektrostatik-GmbH, Weil am Rhein, Germany). The collector consisted of a needle (Sterican^®^, B. Braun, Melsungen, Germany) with the dimensions 0.9 × 50 mm (20 G) and was rotating during the e-spinning process at approximately 120 rpm. The distance between the spinning tip and the collector was adjusted according to the run. The temperature was set at 20 °C. The humidity ranged between 40% and 44%. Electrospun scaffolds were left overnight to evaporate any residual solvent for further analysis.

### 2.6. Fiber Yield Analysis 

The fiber yield obtained on the collector was calculated with the weight of the scaffolds according to following equations:

$$fiber\;yield\left[\%\right]=\frac{quantity\;captured\;by\;collector}{quantity\;applied\;by\;spinning\;nozzle}\times 100$$

$$quantity\;applied\;by\;spinning\;nozzle=flow\;rate[\frac{mL}{h}]\times time\left[h\right]\times PLGA\;solution\left[wt\%\right]$$

The weight was measured using a NewClassic MF scale (Mettler Toledo, Giessen, Germany).

### 2.7. Scaffold Morphology

Characterization of the scaffold was carried out using a digital microscope (VHX-5000, Keyence, Itasca, IL, USA) for precise analysis. Microscope images were acquired at a magnification of 150×. Three specific fixed positions (2 cm, 3 cm, and 4 cm from the edge of the tubular structure) on each scaffold were selected for image acquisition; then, the outer diameter of the scaffold at each of these positions was measured. This approach ensured an evaluation of the scaffold’s structural attributes.

### 2.8. Fiber Morphology (Scanning Electron Microscopy)

The scaffolds underwent sputter-coating (EM SC D500, Leica, Wetzlar, Germany) with a 12.5 nm gold–palladium layer. Afterwards, they were analyzed using scanning electron microscopy (ESEM Quattro S, Thermo Scientific, Darmstadt, Germany) operated at an accelerating voltage of 10.00 kV. The determination of fiber diameter was performed using ImageJ freeware (National Institute of Health, Bethesda, MD, USA) by measuring the diameter of ten randomly chosen fibers per scaffold.

### 2.9. DoE Approach and Statistical Analysis

The factorial design-of-experiments (DoE) method provides a way to produce linear models approximating relationships between input parameters. The correlations between the input and output parameters were investigated. A randomized full factorial design (2^3^) was carried out with Minitab Software (Minitab^®^ 21.4.1). Eight experimental points were investigated, with each point repeated four times. 

The results were analyzed using Minitab Software. Values were calculated by a linear regression fit. The resulting data were presented as the mean ± standard deviation (SD), with DoE comparing four independent experiments and the remaining experiments using three independent experiments as a basis. Group comparisons were conducted using an ANOVA test. Statistical significance was set at *p* < 0.05 unless stated otherwise.

## 3. Results

### 3.1. Disturbance Factors Reduction

**Figure 4:**
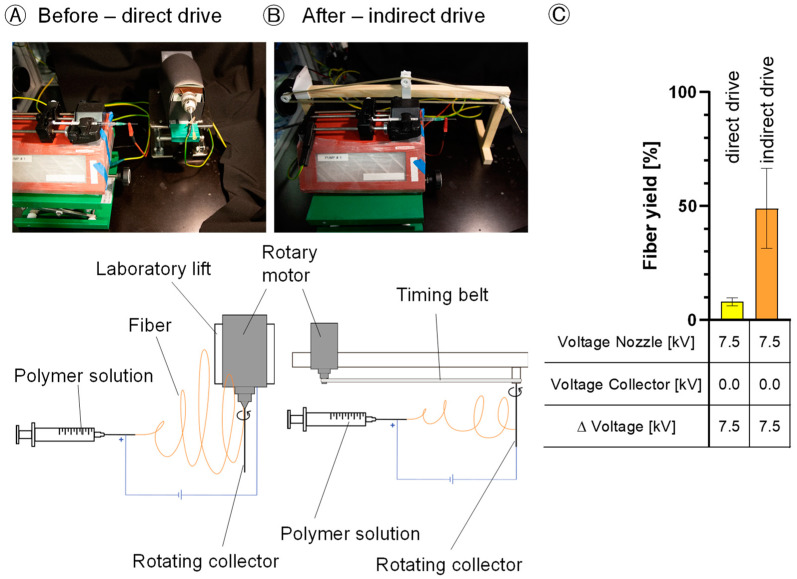
Influence of different setups on the fiber yield on the collector—(**A**): Scheme of the process with a direct rotary drive (before modification); (**B**): Scheme of the process with an indirect rotary drive (after modification); (**C**): Comparison of the mean values of the fiber yield obtained on the collector between setups with a direct drive and indirect drive. Data are shown as the mean of three independent experiments (*n* = 3). *p* < 0.05.

The factors disturbing the electric spinning field were reduced. The relevant factors in this case were the disturbing electrical field of the motor and the influences of the conductive materials of the motor and the laboratory lift which led to fiber deviation from the collector (Figure 4A,B). As a result of this modification, there was a 6-fold increase from 8% ± 1.8 to 49% ± 17.7 in the fiber yield on the collector (Figure 4C).

### 3.2. Pre-Experiments to Evaluate the Process Window for the Design-of-Experiments (DoE) Method

**Figure 5:**
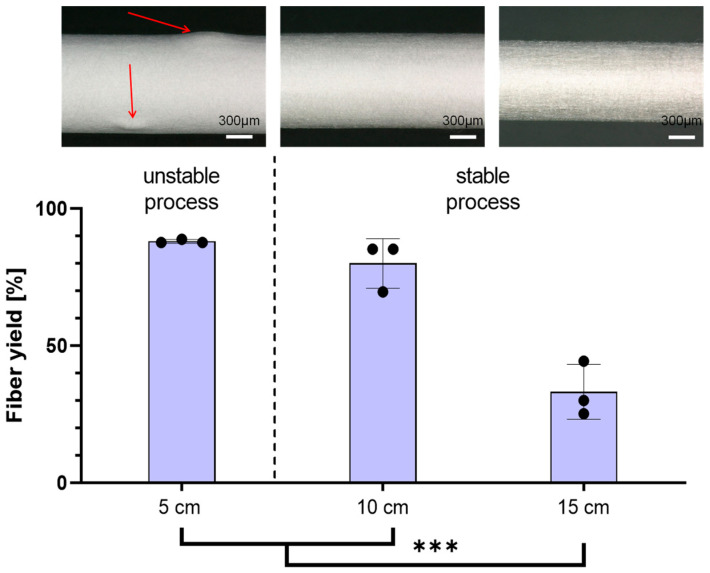
Comparison of fiber yield on the collector with different tip-to-collector distances to define parameters for DoE (voltage spinning nozzle = 6.0 kV/voltage collector = −1.5 kV)—(**Top**): Microscopic picture of scaffolds. Irregular areas (marked with arrows) are present at a distance of 5 cm (**left**) as a result of an unstable spinning process. At 10 cm (**middle**) and 15 cm (**right**), the scaffold is spun evenly; Scale 300 µm; (**Bottom**): Mean values of obtained fiber yield. *n* = 3; *p* < 0.001 (***).

*Tip-to-Collector Distance*: After setup optimization, the tip-to-collector distance was investigated first. Highest fiber yields on the collector were achieved at distances of 5 cm (88% ± 0.7) and 10 cm (80% ± 9.0). The spinning process at 5 cm was reproducible in terms of the fiber yield on the collector; however, it exhibited irregularities and thickening in the scaffold structure (Figure 5, top left). During the spinning process at both the 10 cm and 15 cm (33% ± 10.0) distances, the collector was uniformly covered with fibers (Figure 5, top middle and top right). Increasing the distance from 10 cm to 15 cm resulted in a 47% reduction in the obtained fiber yield on the collector (Figure 5, bottom).

**Figure 6:**
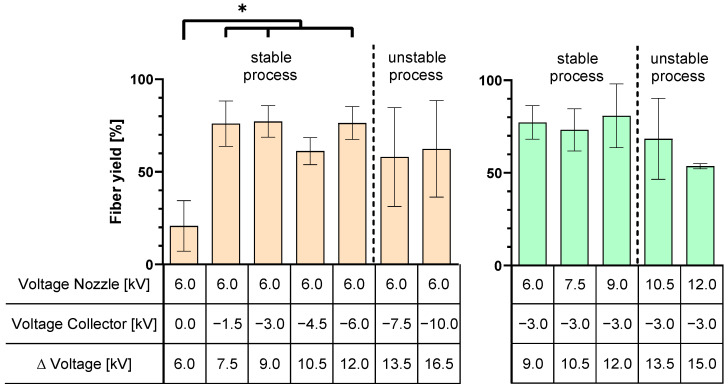
Comparison of different applied voltages on the fiber yield on the collector to define parameters for DoE—left: Variation in negative voltage on the collector; right: Variation in positive voltages on the spinning nozzle (polymer). Data are shown as the mean of three independent experiments. *n* = 3; *p* < 0.05 (*).

*Voltage and Polarity*: An investigation was conducted to determine at which voltages a stable spinning process occurred. When the voltage difference between the spinning nozzle potential and the collector potential exceeded 12 kV, the process became unstable, resulting in the formation of a multi-jet (Figure 6). Applying a negative voltage on the collector significantly increased the fiber yield above 61%, regardless of the voltage level.

### 3.3. Fiber Yield Analysis 

**Figure 7:**
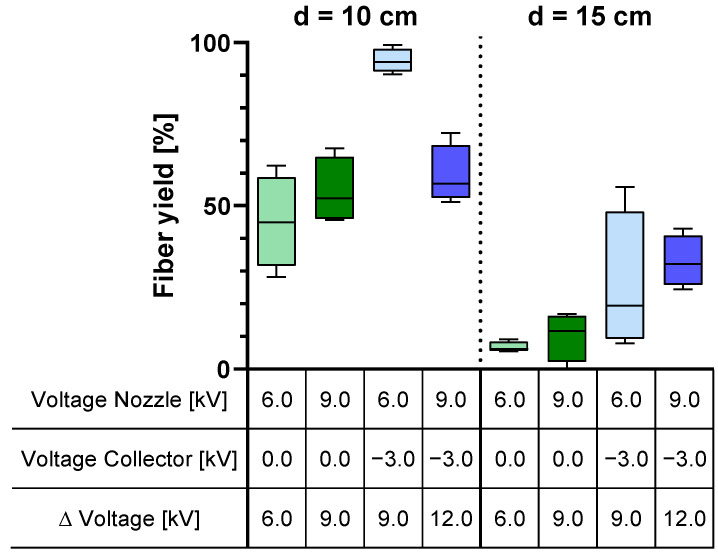
Influence of applied potential and tip-to-collector distance (d) on the fiber yield on the collector; *n* = 4.

The efficiency of the electrospinning process was investigated in order to optimize the manufacturing process and minimize material loss. Therefore, the fiber yield on the collector was determined. A maximum yield of 94% ± 3.7 could be achieved with an applied voltage of 6.0 kV at the spinning nozzle and −3.0 kV at the collector (Figure 7). Increasing the spinning nozzle voltage (collector voltage = −3 kV) results in a highly significant decrease in fiber yield on the collector to 59% ± 9.1 at a distance of 10 cm (*p* = 0.0032). Expanding the tip-to-collector distance reduces the efficiency of the e-spinning process (Figure 7). In comparison to the previous e-spinning setup with a direct rotation drive described in Chapter 3.1, the fiber yield on the collector was increased by a factor of 12 from 8% ± 1.8 to 94% ± 3.7.

### 3.4. Scaffold Morphology

**Figure 8:**
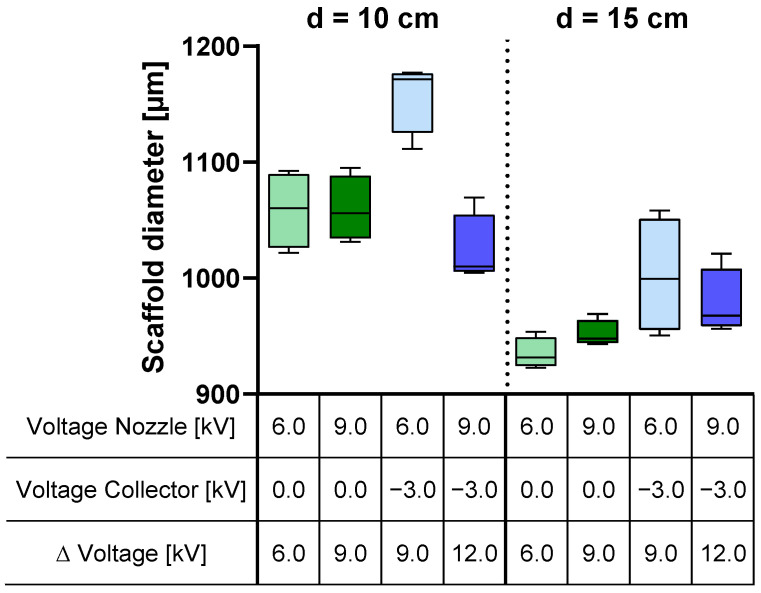
Influence of the applied potential and tip-to-collector distance (d) on the scaffold diameter; collector diameter: 900 µm; *n* = 4.

Investigation on the outer diameter of the scaffold shows similar results to the analysis of the fiber yield on the collector (refer to Figure 7 and Figure 8). Increasing the spinning nozzle voltage with applied negative voltage results in a significant decrease in the scaffold diameter (from 1158 µm ± 31.2 to 1023 µm ± 30.8) (*p* < 0.001). Widening the tip-to-collector distance reduces the scaffold thickness (Figure 8). The measured outer diameters of the scaffolds were taken at three different points, corresponding to scaffold lengths of 2 cm, 3 cm, and 4 cm. No significant differences between the three measuring points were observed within the scaffolds.

### 3.5. Fiber Morphology 

**Figure 9:**
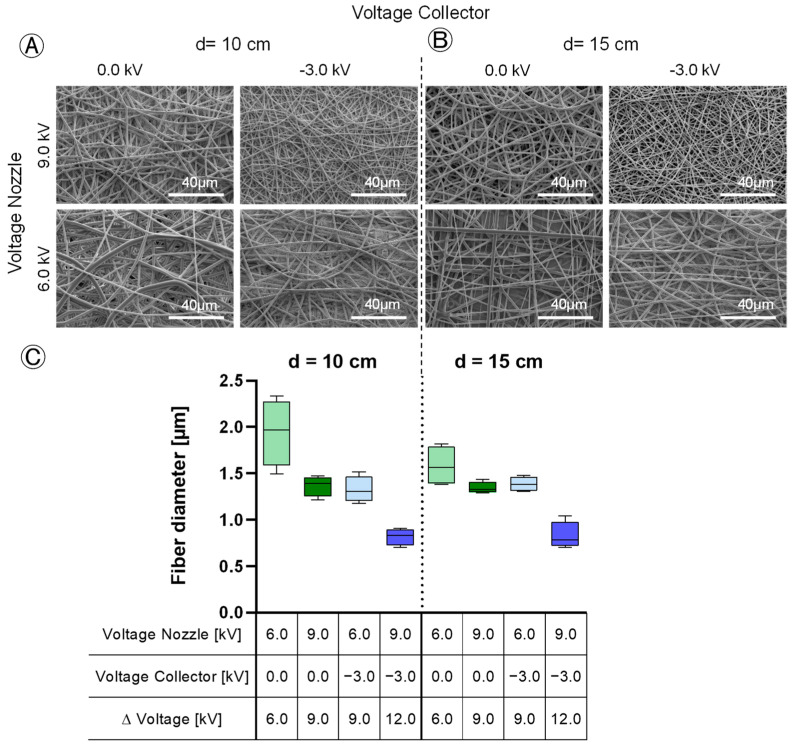
Influence of applied voltages on fiber diameter—(**A**,**B**): SEM micrographs of scaffolds with the tip-to-collector distance (d) = 10 cm (**A**) and d = 15 cm (**B**), Scale: 40 µm; (**C**): Mean value of fiber diameters of all scaffolds; *n* = 4.

The influence of the applied voltages and the tip-to-collector distance on the fiber diameter and variation was investigated. The increase in the electric field leads to a reduction in the fiber diameter (from 1.943 µm ± 0.359 to 0.817 µm ± 0.090 at 10 cm distance and from 1.580 µm ± 0.215 to 0.828 µm ± 0.147 at 15 cm distance) (Figure 9C). At low voltage differences between the spinning nozzle and collector, the fiber diameter shows significant inhomogeneity with differences of max. 3.23 µm (*p* = 0.001) (Figure 9A,B). At a difference of 6.0 kV, the thickest fibers up to 3.70 µm ± 0.663 are spun, the diameter of the thinnest fibers in the same samples was 0.47 µm ± 0.091. With a voltage difference exceeding 9.0 kV, the spinning process becomes more homogeneous, leading to more uniform fiber diameters. The absolute voltages have no significant effect, only the potential difference affects the fiber diameter. No reduction in fiber diameter was observed with an increasing tip-to-collector distance (Figure 9C). Bead formation was not detected in any of the experiments (Figure 9A,B). 

### 3.6. Effect Analysis

**Figure 10:**
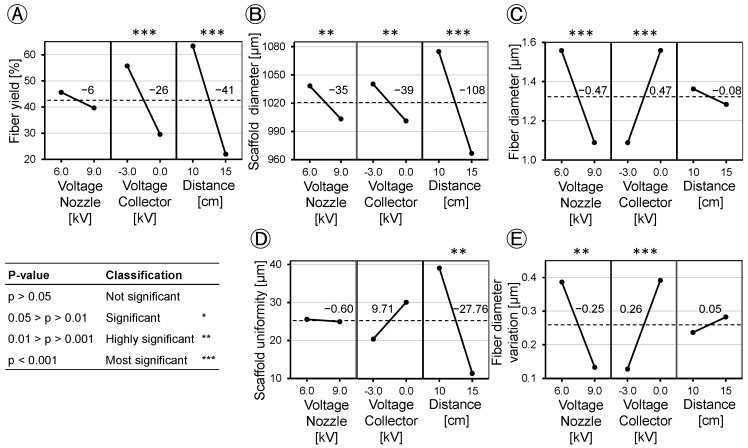
Main effects of applied voltages and tip-to-collector distance (*n* = 4). (**A**): Fiber yield; (**B**): Diameter scaffold; (**C**): Fiber diameter; (**D**): Scaffold uniformity; (**E**): Fiber diameter variation.

**Figure 11:**
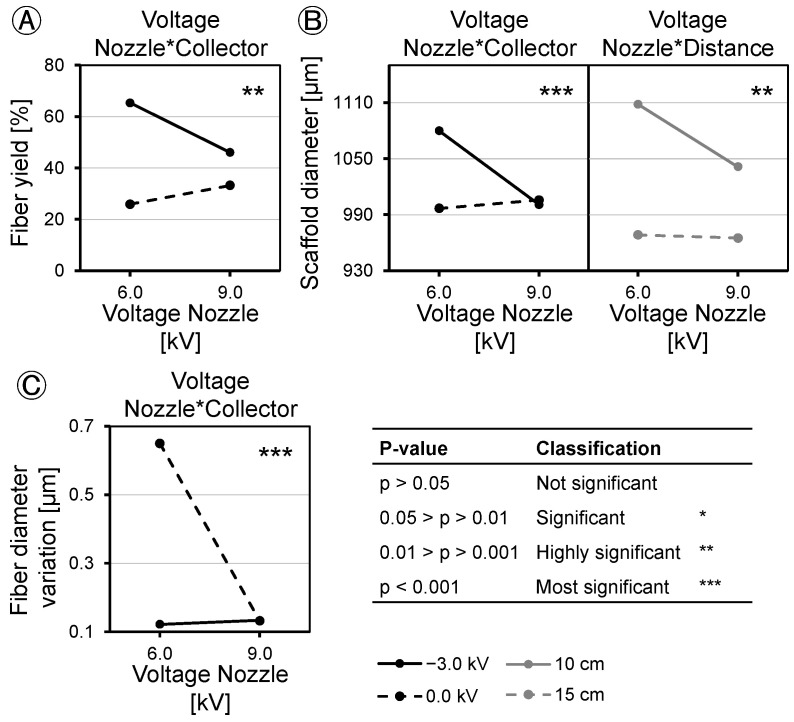
Interaction diagrams showing statistically significant interactions on (**A**): Fiber yield on the collector, (**B**): Scaffold diameter; (**C**): Fiber diameter variation. Non-represented interactions do not have significant influences on output parameters (*n* = 4).

Based on the DoE method, we analyzed the applied voltage and tip-to-collector distance and found different effects on fiber yield, scaffold diameter, scaffold uniformity, fiber diameter, and fiber diameter variation (Figure 10). Concerning fiber yield, the effect of spinning nozzle charging was found to be statistically insignificant (*p* = 0.124). Applying a charge of −3.0 kV to the collector increased the fiber yield on the collector by 26% at *p* < 0.001. Increasing the distance from 10 cm to 15 cm led to a decrease in fiber yield by 41% at *p* < 0.001 (Figure 10A). When the collector was grounded, raising the spinning nozzle voltage resulted in an increased fiber yield of 19%, whereas increasing the spinning nozzle voltage under a negative voltage on the collector led to a decrease of 7% in fiber yield at *p* = 0.002 (Figure 11A). Spinning nozzle voltage alone did not show a statistically significant effect, but it demonstrated interactions with the collector voltage.

Evaluation of the scaffold diameter revealed similar effects for the applied voltages on the spinning nozzle (−35.08 µm at *p* = 0.004) and the collector (−39.08 µm at *p* = 0.001), while the distance between the spinning nozzle and the collector exerted a threefold greater influence on the scaffold diameter (−108.08 µm at *p* < 0.001) (Figure 10B). The scaffold diameter showed minimal influence of nozzle charging when no collector voltage was applied (8.92 µm at *p* < 0.001). It increased about 79.08 µm at a *p* < 0.001 when the spinning nozzle voltage was reduced, coupled with a collector voltage of −3.0 kV (Figure 11B, left). The spinning nozzle voltage also interacted with distance concerning the scaffold diameter. No discernible influence was noted with changing nozzle charging at a distance of 15 cm (−3.33 µm at *p* = 0.008). When the distance of the spinning nozzle and the collector was 10 cm and spinning nozzle voltage was reduced, the scaffold diameter increased by about 66.83 µm at *p* = 0.008 (Figure 11B, right).

The tip-to-collector distance emerged as the only significant factor influencing scaffold uniformity (Figure 10D). At a 10 cm distance, a more pronounced deviation in the outer diameter of the scaffold (39.135 µm) was observed compared to that at a 15 cm distance (11.380 µm) at *p* = 0.001. Other factors did not show any significant interactions with each other, concerning scaffold uniformity.

The study investigated fiber diameter and found contrasting effects resulting from changes in the spinning nozzle voltage and the collector voltage, both of which had a notably significant effect (*p* < 0.001). Increasing the spinning nozzle voltage from 6.0 kV to 9.0 kV led to a decrease in fiber diameter by 0.47 µm. However, the tip-to-collector distance did not show a significant effect on fiber diameter (*p* > 0.05) (Figure 10C). At the maximum fiber yield on the collector of 94% ± 3.7, fibers with an average diameter of 1.3 µm ± 0.141 were produced. When thinner fibers were desired for the scaffold composition, it led to a decrease in the fiber yield. The factors did not show any interaction with each other with respect to the fiber diameter. The variation in the fiber diameter is dependent on the field strength of the e-spinning field. Similar to the fiber diameter, at −0.25 µm (*p* = 0.001) with increasing nozzle voltage and 0.26 µm (*p* < 0.001) with increasing collector voltage, the effects of the fiber diameter variation were opposite but equally pronounced (Figure 10E). A higher field strength resulted in the production of more uniformly spun fibers. The tip-to-collector distance did not significantly affect the variation in the fiber diameter. The interaction between the individual voltages was also visible from the interaction diagram (Figure 11C). Increasing the nozzle voltage with a negatively charged collector has a minimal effect of 0.01 µm on the fiber diameter variation; with a grounded collector, the fiber diameter variation decreases by 0.52 µm as the nozzle charge increases (*p* < 0.001).

### 3.7. Electrospinning of Drug-Loaded Scaffolds

**Figure 12:**
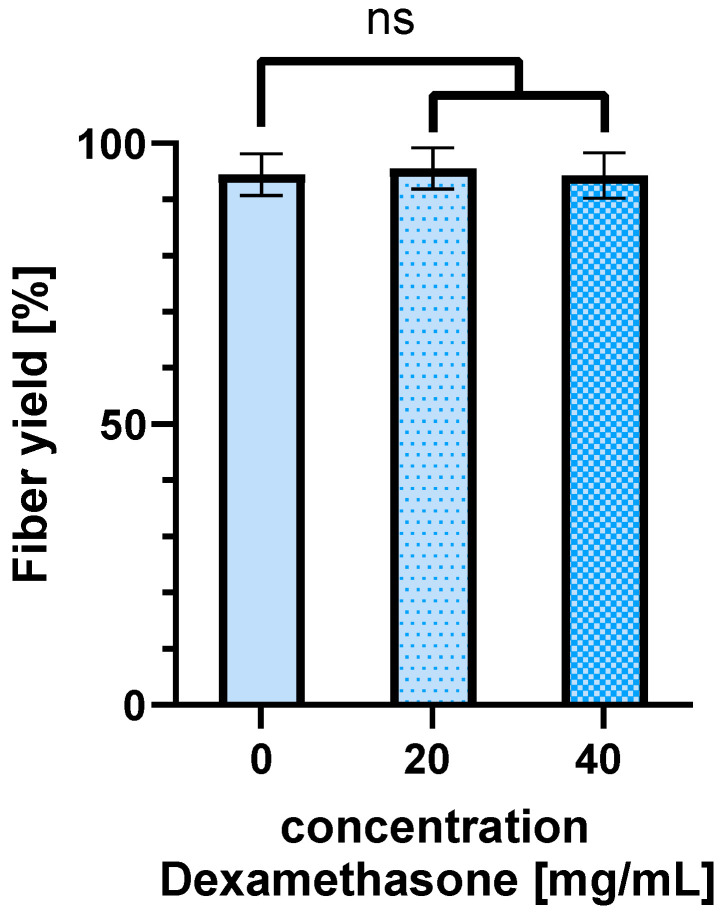
Obtained fiber yield on the collector while integrating different concentrations of Dexamethasone (20 mg/mL; 40 mg/mL) in the spinning process. No significant difference in fiber yields could be observed. Spinning parameters: voltage nozzle = 6.0 kV; voltage collector = −3.0 kV; tip-to-collector distance = 10 cm; *n* = 4; not significant (ns) *p* > 0.05.

With the optimal parameters obtained in this DoE study (voltage nozzle: 6.0 kV, voltage collector: −3.0 kV, tip-to-collector distance: 10 cm), we spun in different concentrations of Dexamethasone (20 mg/mL; 40 mg/mL) to determine whether this has an influence on the fiber yield obtained on the collector. Compared to the fiber yield of the spinning process without the drug Dexamethasone (94% ± 3.7), a fiber yield of 95% ± 3.7 was achieved by spinning a 10 (m/V)% PLGA solution with 20 mg/mL dexamethasone. At 40 mg/mL Dexamethasone in the 10 (m/V)% PLGA solution, a fiber yield of 94% ± 4.0 was obtained. There is no significance (ns) (0 mg/mL dexamethasone vs. 20 mg/mL dexamethasone *p* = 0.914; 0 mg/mL dexamethasone vs. 40 mg/mL dexamethasone *p* = 0.998) (Figure 12).

## 4. Discussion

Tissue engineering uses electrospinning for its porous structure, which mimics the extracellular matrix and facilitates the integration of bioactive substances. However, the fabrication of sub-millimeter tubular structures is challenging because disturbances affect the fiber yield. This study aims to optimize electrospinning for small tubular structures (I.D. < 1 mm) by removing disturbances, analyzing control variables, and evaluating process parameters using PLGA as a model polymer. The results show significant improvements in fiber yield of up to 94% and in scaffold morphology, providing insight into the cost-effective production of small tubular structures.

The reduction in factors disturbing the electrical field is a critical factor in the successful implementation of an e-spinning process. Disturbances of the electrical field have a significant effect on the (re)producibility and quality of the resultant fibers. Eliminating external disturbances, such as the rotational motor and laboratory-lifting platform [24], ensures a precise fiber alignment towards the collector, resulting in a 6-fold increase in fiber yield and facilitating the application of a negative voltage for a more targeted trajectory for the electrospun fibers. 

Following the optimization of the experimental setup, an investigation was conducted to define the process window for the DoE. In our study, we opted for the factors of the tip-to-collector distance, spinning nozzle voltage, and the voltage applied on the collector, as they have a major impact on the e-spinning process. Initially focusing on the tip-to-collector distance, it was revealed that the highest fiber yields with over 80% on the collector were obtained at distances of 5 cm and 10 cm. At 5 cm, while the fiber yield was reproducible, it led to irregular and thickened areas in the scaffold structure. The process was described as unstable as the spinning was uneven. Due to the short distance between the e-spinning nozzle and the collector, droplets are drawn out of the spinning nozzle and propelled directly towards the collector without descending to the ground. Shortening the distance between the spinneret and the collector reduces the evaporation time of the solvent, which can lead to poor fiber formation and a reduction in fiber stability and clumping of the fibers [25]. Comparatively, the spinning process at both 10 cm and 15 cm distances uniformly covered the collector with fibers and was therefore labeled as a stable spinning process. However, increasing the distance from 10 cm to 15 cm resulted in a notable 38% reduction in the obtained fiber yield on the collector. Due to the increased distance between the spinning nozzle and the collector, the force with which the fibers are pulled to the collector is weakened. External interferences now exert a greater influence on the fibers, causing them to deviate from the collector. In the setup used with the polymer solution used, the optimum distance between the spinneret and the collector is 10 cm.

The investigation of the voltages required for a stable e-spinning process showed that a voltage difference of more than 12 kV between the spinning nozzle potential and the collector potential leads to the formation of a multi-jet, which makes the process unstable. The presence of this unstable multi-jet resulted in increased variance in the collected fiber yield.

In order to optimize the manufacturing process and reduce material wastage, an analysis of the e-spinning process efficiency was carried out, with a focus on the fiber yield on the collector. It was observed that a peak yield of 94% was achieved with an applied voltage of 6.0 kV at the spinning nozzle and −3.0 kV at the collector. Increasing the spinning nozzle voltage resulted in a 42% reduction in fiber yield on the collector, as the heightened voltage difference accelerated the fibers, causing them to bypass the collector. Furthermore, an increase in the tip-to-collector distance was found to lower the efficiency of the e-spinning process, confirming the findings of Tomaszewski and Szadkowski [2]. Due to the increased distance between the nozzle and the collector, the force pulling the fibers towards the collector decreases. Disturbance factors deflect the fibers, resulting in a loss of material on the equipment, consequently reducing the fiber yield on the collector significantly. Comparatively, our modified e-spinning setup with an indirect rotation of the collector, in contrast to the prior configuration with a direct rotation drive, yielded a 12-fold increase in fiber yield on the collector. Low fiber yields on the collector not only lead to material loss and, consequently, an increase in process costs (especially with expensive educts and when proteins are encapsulated), but also result in the contamination of the device and spinning equipment.

The examination of the outer diameter of the scaffold demonstrated a parallel trend to the analysis of the fiber yield on the collector, as depicted in Figure 7 and Figure 8. This correspondence can be attributed to the direct influence of higher fiber yields on the collector, which consequently led to a larger diameter of the resulting scaffold. This relationship highlights the critical role of process parameters in determining the final scaffold geometry, where a higher fiber yield contributes to an increased scaffold thickness while maintaining a constant spinning time. 

Applying a high voltage to the spinning nozzle held by its surface tension to the tip generates a charge on the liquid’s surface. The mutual repulsion of these charges and the convergence of surface charges towards the counter electrode create a force that acts directly against the surface tension. As the electric field intensity increases, the hemispherical drop formed at the tip of the needle transforms into a conical shape, a phenomenon known as the Taylor cone [26]. As the electric field is increased, the force with which the fiber is drawn also increases, leading to a reduction in the fiber diameter [27]. Smaller electric fields can result in inhomogeneous fields, leading to varying impacts on the polymer flows and thus the fiber formation. Consequently, when the voltage differences between the spinning nozzle and the collector are low, the fiber diameter shows significant inhomogeneity. Moreover, in the case of low electric fields, insufficient fiber stretching can also result in a wider distribution of fiber diameters. Beyond a 9.0 kV voltage difference, the spinning process becomes more homogeneous, resulting in more uniform fiber diameters. This study shows that only the voltage difference influences the fiber diameter, while the distribution of how the voltages are applied to the spinning nozzle and collector seems not to play a role. However, in contrast to Kalluri et al. [22] and Ostheller et al. [28], no reduction in fiber diameter was observed with an increase in the tip-to-collector distance. Although an increase in distance results in a weakening of the electric field, it appears to be insufficient to significantly change the fiber diameter. 

The application of DoE to analyze the applied voltage and tip-to-collector distance revealed variations in their effects on fiber yield, scaffold diameter, scaffold uniformity, fiber diameter, and fiber diameter variation. Regarding fiber yield, nozzle charging has no significant effect. However, charging the collector with −3.0 kV increases the fiber yield on the collector by 26%. On the other hand, increasing the distance from 10 cm to 15 cm decreases the fiber yield by 41%. This implies that an increase in yield is attainable with negative collector charging and a working distance of 10 cm. When a negative voltage is applied, the fibers are directed towards the collector. However, if the distance between the nozzle and the collector becomes too large, environmental disturbances have a greater influence, causing the fibers to disperse within the device and on the instruments. Factors can interact with each other, and in the case of the fiber yield, the factors of spinning nozzle voltage and collector voltage show such an interaction. Increasing the spinning nozzle voltage with a grounded collector results in an increase in the fiber yield, and the fiber yield decreases when the nozzle voltage is increased with a negative voltage applied to the collector. It should be noted that the nozzle voltage alone does not have a statistically significant effect, but it interacts with the collector voltage. 

When comparing the influence of voltages and distance with the trends observed in the mean values of fiber yield and scaffold diameter (refer to Figure 4 and Figure 5), one might assume that the effects on both are similar. In assessing the scaffold diameter, aside from the yield, the applied voltages demonstrated similar effects, while the distance between the spinneret and the collector exhibited a threefold greater influence on the scaffold diameter. These disparities in the effects on yield and scaffold diameter can be explained by variations in the packing density of the fibers, resulting in differing levels of scaffold porosity. Scaffolds with small pore sizes but the same mass compared to scaffolds with large pore sizes exhibit a reduced outer diameter. In contrast to the fiber yield, the effects interact more strongly with each other. While the scaffold diameter is hardly influenced when no collector voltage is applied, it increases when the spinning nozzle voltage is reduced and a collector voltage of −3.0 kV is applied. Nozzle voltage also interacts with distance regarding the scaffold diameter. Similar to the previous interaction, there is no influence when the collector is grounded. However, when the collector is negatively charged and the spinning nozzle voltage is reduced, the scaffold diameter increases.

The tip-to-collector distance is the only significant factor affecting scaffold uniformity. At a 10 cm distance, there is greater deviation in the outer diameter of the scaffold compared to a 15 cm distance. This can be attributed to fewer fibers landing on the collector at 15 cm, where the width of the collector becomes more significant. Larger mass depositions of fibers on the collector at 10 cm have a more pronounced impact on scaffold uniformity. The factors do not interact with each other for scaffold uniformity.

Furthermore, the study examined the fiber diameter, revealing opposing effects from changes in the spinning nozzle voltage and the collector voltage, both of which had a comparably significant impact. Increasing the spinning nozzle voltage from 6.0 kV to 9.0 kV led to a reduction in the fiber diameter by 0.47 µm. The fiber diameter is influenced by the field strength of the e-spinning field (difference between the spinning nozzle and collector voltage), but not by the individual voltages themselves. The higher the field strength, the more the Taylor cone is stretched, and the fibers speed up, resulting in a thinner fiber diameter. The tip-to-collector distance was found to have no significant effect on the fiber diameter. When maximizing the fiber yield on the collector, a compromise must be made with the fiber diameter. At the maximum fiber yield on the collector of 94%, fibers with an average diameter of 1.4 µm are spun. If finer fibers are desired to compose the scaffold, it results in a reduction in the fiber yield. The factors do not interact with each other in terms of the fiber diameter. The variation in the fiber diameter is also dependent on the field strength of the e-spinning field. Therefore, as with the fiber diameter, the effects are opposite but equally pronounced. A higher field voltage results in more uniform fibers being spun. In this regard, the individual applied voltages do not play a role, only the difference between the spinning nozzle and the collector voltage matters. The tip-to-collector distance has no significant influence on the fiber diameter variation. The interaction between the individual voltages is also visible from the interaction diagram. In this context, increasing the spinning nozzle voltage stabilizes the fiber diameter more when the collector is grounded than when it is charged. The reason for this is that the process is already more stable when the collector is negatively charged, leading to even spun fibers. 

Electrospun scaffolds can be used as drug delivery systems. In this process, drugs are spun into the fibers. This study shows that the loading of the scaffold with the demonstrator drug Dexamethasone in different concentrations has no significant effect on the fiber yield obtained on the collector. By using different polymers, the release of the drug can be adjusted as needed. It should be noted that changing the polymer changes the chemical properties of the spinning solutions. This affects the spinnability of the polymer. However, the basic parameters for the spinning process are similar. Therefore, the basic relationships can be transferred to other polymers, but fine-tuning of the parameters will certainly be necessary. This manuscript provides guidance on how the DoE method can be used to achieve an optimal process with comparatively little effort, even with other polymers.

## 5. Conclusions

In conventional e-spinning setups, the fiber yields on the collector in the sub-millimeter level range from 5% to 15% due only to the massive loss of material on non-collector surfaces. As the incorporation of drugs and bioactive substances is more and more important and can be very expensive (1000 to 20,000 EUR per milligram), this low fiber yield is very problematic from a production point of view. In this study, we optimized the e-spinning process for sub-millimeter tubular scaffolds to enhance manufacturing efficiency and minimize material loss. The knowledge gained now makes it possible to efficiently spin higher value, more expensive materials such as recombinant proteins, growth factors, or doped materials. The results of this study are not only important for tissue engineering, but also for other applications such as the production of membranes or hollow fibers. The results revealed that adjusting the applied voltage and specific configurations on the collector notably influenced fiber yield, with a peak yield of 94% observed using PLGA as the model polymer. The understanding of the complex relationships between the process parameters can optimize e-spinning for the cost-effective production of tissue-engineered tubular scaffolds with sub-millimeter inner diameters. Additionally, the findings have broad implications for diverse scientific fields, including nerve tissue engineering and membrane production. The ability to customize scaffold properties through precise process control opens avenues for advanced material development across scientific, medical, and industrial domains.

## Figures and Tables

**Table 1:** Output parameter of DoE.

Response	Parameter	Unit	Method
y_1_	Fiber yield	%	Weighting mass deposition
y_2_	Scaffold diameter	µm	Optical microscopy
y_3_	Uniformity scaffold diameter	µm	Optical microscopy
y_4_	Fiber diameter	µm	SEM
y_5_	Fiber diameter variation	µm	SEM

**Table 2:** Classification of electrospinning parameters, gray highlighted factors chosen for study.

Classification	Process Parameter
**Disturbance factor**	Device design (setup/size/material of the spinning chamber; disturbance of the electrical field by chamber design and electric components)
	Operator (preparation of solution and machine operation)
	Batch variation
	Chemical contamination
**Control factor**	Δ Voltage potential between the nozzle and the collector
	Collector charging (grounding vs. negative vs. positive charge)
	Tip-to-collector distance
	Collector size and geometry
	Spinning time
	Flow rate of the spinning solution
	Humidity
	Temperature
	Solvent (concentration, viscosity, evaporation rate, etc.)
	Properties of the spinning solution (concentration, viscosity, salinity, etc.)
	Nozzle diameter
	Polarity of the spinning solution

**Table 3:** Input parameter for a 2 k full factorial DoE (k = 3).

Factors	Parameter	Unit	Low Level (−1)	High Level (+1)
k_1_	Voltage nozzle	kV	6.0	9.0
k_2_	Voltage collector	kV	−3.0	0.0
k_3_	Tip-to-collector distance	cm	10.0	15.0

## Data Availability

All data that support the findings of this study are included within the article.
